# Awareness, attitudes, and beliefs of dementia in Indonesia

**DOI:** 10.1002/dad2.12570

**Published:** 2024-04-12

**Authors:** Nicolas Farina, Esra Hassan, Imelda Theresia, Fasihah Irfani Fitri, Ika Suswanti, Tara Puspitarini Sani, Sara Evans‐Lacko, Sube Banerjee, Yuda Turana

**Affiliations:** ^1^ Faculty of Health University of Plymouth Devon UK; ^2^ Centre for Dementia Studies, Brighton and Sussex Medical School East Sussex UK; ^3^ Alzheimer Indonesia Jakarta Indonesia; ^4^ Department of Neurology, Faculty of Medicine Universitas Sumatera Utara Medan Indonesia; ^5^ Department of Neurology School of Medicine and Health Sciences Atma Jaya Catholic University of Indonesia Jakarta Indonesia; ^6^ Care Policy and Evaluation Centre London School of Economics and Political Science London UK; ^7^ Faculty of Medicine and Health Sciences University of Nottingham Nottingham UK; ^8^ School of Medicine and Health Sciences Atma Jaya Catholic University of Indonesia Jakarta Indonesia

**Keywords:** attitudes, beliefs, general public, Indonesia, knowledge, stigma

## Abstract

**INTRODUCTION:**

Tackling dementia stigma is a policy priority. In Indonesia, we have little insight into the general public's knowledge and attitudes about dementia.

**METHODS:**

Cross‐sectional study of 4430 Indonesian adults recruited from Jakarta and North Sumatra, Indonesia. Measures included dementia knowledge and attitudes.

**RESULTS:**

A total of 86.3% (*n* = 3,803) of adults had not heard of the terms dementia or Alzheimer's disease, and commonly viewed dementia as a normal part of aging. Being older, incorrect knowledge about etiology, not having heard of the terms dementia and/or Alzheimer's disease, having less than primary education, and being from North Sumatra were associated with more negative attitudes (*p*‐values < 0.05).

**DISCUSSION:**

Misconceptions and lack of awareness about dementia are common in Indonesia. Attitudes tended not to be negative, but our research highlights factors associated with dementia attitudes. Future research should use this information to better tailor and target potential anti‐stigma strategies.

**Highlights:**

1. Most Indonesians had not heard of the terms dementia and/or Alzheimer's disease and thought it was caused by normal aging.
2. The majority of participants held mixed or positive attitudes towards dementia.
3. A series of demographic factors alongside poor awareness were associated with negative attitudes towards dementia.

## 1 BACKGROUND

Over 57.4 million people currently worldwide are estimated to have dementia.^1^ Low and middle–income countries (LMICs) are projected to see the greatest increase in dementia prevalence due to increased life expectancy.^2^ Indonesia is a lower middle‐income country, and current estimates suggest that it has close to 1 million people with dementia,^1^ although this figure could be higher with many going undiagnosed.^3^

Stigma can have a profound impact on the lives of people living with the condition.^4^, ^5^ Conceptually, there are different types of stigma.^6^ In this article we focus on “public stigma” which concerns the general public as perpetrators.^7^ The term public stigma encompasses subdomains such as misinformation (knowledge), prejudice (attitudes), and discrimination (behavior).^6^ Public stigma can be described as a collection of negative attitudes and beliefs that lead to discrimination and avoidance behaviors toward a group of people.^8^ Internationally, a small but significant group hold negative attitudes and beliefs toward people with dementia.^4^ However, it is important to recognize that what stigma looks like varies between and within countries, with culture playing an important role in how it forms.^9^, ^10^, ^11^

Public awareness campaigns, education, and contact, are often seen as the key strategies to tackle stigma.^12^ The Indonesian government has adopted a national dementia plan, in which raising public awareness is a key priority.^13^ The extent to which these campaigns have been rolled out, and their success is less clear. However, to tailor public awareness campaigns, it is important to target the “who” but also “what” needs to be changed.^14^ Misconceptions that dementia is a normal part of aging, a common view held internationally,^4^ may shape risk reduction and help‐seeking behaviors.^15^ Although gaps in knowledge may not be universal, lower education and being male are associated with poorer dementia knowledge.^16^ As such, adopting a “one‐size fits all” approach is unlikely to be the most effective way to tackling stigma. Within Indonesia, there is an apparent gap in our knowledge about what public stigma looks like, thus limiting our ability to develop effective stigma reduction strategies.

In Indonesia, very little research has explored attitudes and beliefs of dementia among the general public. In one report, 44.1% of Indonesians (sample size not described) reported that they believed that people with dementia are impulsive and dangerous.^4^ In a more localized sample from Yogyakarta (*n* = 203), the authors describe that the participants views were “mostly pessimistic” about Alzheimer's disease.^17^ In terms of who is at risk of holding these attitudes and beliefs, only a single study has explored such associations within Indonesia.^17^ The authors reported a positive association between age and attitudes (*r* = 0.18, *p* = 0.01), but there were no statistically significant associations reported with sex, experience of dementia (family or via seminar), or dementia knowledge.

**RESEARCH IN CONTEXT**

1. **Systematic review**: The authors reviewed the literature using scientific databases (e.g., Google Scholar and SCOPUS). The extent to what dementia stigma looks like in low‐ and‐ middle income countries is limited, and there are only two studies derived from Indonesia.
2. **Interpretation**: Terms such as Alzheimer's disease and dementia were not widely known among the Indonesian sample. People who had not heard of the terms, were associated with incorrect beliefs about etiology and generally had poorer attitudes. For the most part, people held mixed or positive attitudes toward dementia.
3. **Future directions**: Evidence‐based anti‐stigma initiatives need to be developed for use in Indonesia. Raising awareness of dementia, tailored to specific demographics, may prove to be the most effective strategy.

These findings seem to contradict the broader literature on dementia stigma, which suggests that factors influence stigmatizing attitudes such as age, gender, personal experience, ethnicity, culture, understanding of prognosis, and experience with persons living with dementia.^18^ In addition, the extent to which the current Indonesian findings are generalizable is unclear as these studies adopted either an opportunistic recruitment strategy,^4^ or were already engaged in a free dementia seminar.^17^

To ensure we are able to develop dementia stigma reduction strategies tailored to Indonesia, we need to generate better quality and more representative data. Understanding how dementia knowledge and attitudes might differ across a population also provides more nuance in how we best adapt stigma reduction strategies to different groups. The aim of this study was to develop a better understanding about the knowledge and attitudes of dementia in a representative sample of Indonesian adults, while also ascertaining whether stigmatizing beliefs differ based on existing knowledge, but also age, sex, education attainment, and region.

## 2 METHODS

The study utilizes data from the STrengthening Responses to Dementia in dEveloping countries (STRiDE) study on dementia prevalence within Indonesia. The overarching methodology can be read in detail elsewhere.^3^, ^19^

### 2.1 Participants

Recruitment occurred within Jakarta and North Sumatra. Older adults (aged 65 years and older) were listed following the random selection of districts and subdistricts within each site. A database of older adults within in each cadre (listed July–August 2021) was extracted and randomized to form a list of people to approach. We sampled the number of older adults proportionate to the size of the region where they were listed. If there were multiple older adults within the household, then only one older adult was selected.

All participants were required to speak Bahasa Indonesian and have an informant (e.g., family member, friend) that could also participate. Potential participants were excluded if they resided in care or nursing homes, or lacked capacity to consent, and could not identify a personal consultee to assist in the consent process. A total of 2110 older adults (i.e., 4220 participants when including the informant) were recruited and had sufficient data for primary analysis (Jakarta, *n* = 2114; North Sumatra, *n* = 2108).^3^ For this paper, we excluded participants who identified that they had previously been diagnosed with dementia (or their informant) (*n* = 10).

### 2.2 Procedures

Following the random identification of older adults using existing registers, researchers visited potential participants’ homes (or another location convenient to participants) in pairs. Researchers would then confirm eligibility, including the availability of the informant, prior to obtaining informed consent. If the participant was ineligible or refused, the researchers would move to the next name on the list of older adults.

Informed consent was obtained (written or oral) from the older adult and an identified informant. Participants then completed a series of questions related to identifying cases of dementia and to understand how this may impact the lives of people's lives. The focus of this study will be on stigma‐related outcomes. These items were consistently placed toward the end of toolkit. For the dementia stigma items, the older adult and informant were asked to self‐report on the questions independently. The questionnaire was administered by a researcher and data were collected within the REDCap data capture tools (REDCap Consortium, Vanderbilt University, Nashville, TN, USA) hosted at the London School of Economics and Political Sciences.^20^

The researchers across both sites were provided standardized training and had access to standardized operating procedures prior to testing. Data collection occurred between September 2021 and December 2021.

### 2.3 Measures

All participants (older person and informant) were asked a standard set of socio‐demographic questions including age, sex, and education. The dementia stigma measure comprised the following elements:

1. Awareness of dementia terms. A multiple‐choice question related to whether the participant has heard of the terms “dementia” (*demensia*) or “Alzheimer's disease” (*Penyakit Alzheimer*).
2. All participants were asked about terminology they would use associated with a brief description of dementia: “What word or words would you use to describe an older adult experiencing memory loss and difficulties with thinking, problem‐solving, and language, so much so it affects their ability to perform everyday activities?”^a^
3. Etiology beliefs were captured through 12 multi‐response items spanning a range of accurate (e.g., a brain disease) and inaccurate causes (e.g., witchcraft) of dementia. Two additional items include “other” and “don't know.” Items were taken and adapted from the World Alzheimer's Report 2019.^4^
4. Items from the World Alzheimer's Report 2019 stigma survey^4^ were utilized to capture dementia attitudes and beliefs. Items reflected beliefs about prognosis, diagnosis, and ability to live well with dementia, societal views, and behavioral intentions (e.g., help seeking).

The process of translating and cross‐culturally adapting the measures into Bahasa Indonesian is reported elsewhere.^19^, ^21^

### 2.4 Analysis

We did not distinguish between the older adult and informant in the analysis; hence, the cohort includes both a random sample of older adults and convenience sample of informants (not restricted by age). Demographics were initially reported for people who had heard of dementia and/or Alzheimer's before, and those who had not. Between group comparisons were made (e.g., *t*‐tests).

Open text responses were collected from participants related to terminology they would use to describe someone with dementia. Clear typographical errors were corrected. Each response was translated into English, independently by an Indonesian and English speaker. Valid terms were counted, then grouped into similar themes akin to content analysis. Responses that included multiple terms than spanned multiple categories were flagged as such, so not to inflate any individual category through “double counting.”

To understand how having general awareness influenced etiology beliefs, odds ratios (and 95% confidence intervals [CIs]) were generated between dementia awareness (1 = heard of Alzheimer's or dementia) and individual etiology outcomes. Subsequently, the analysis was adjusted for age, sex (1 = Male), education attainment (1 = completed primary school or higher), and site (1 = North Sumatra).

Macdonald's Omega was calculated on all attitude items to ascertain unidimensional reliability (95% bootstrapped CIs, 1000 bootstrap samples).^22^ Macdonald's Omega represents the extent to which the total score provides a reliable measure of the underlying factor structure, and is often considered a more robust measure of reliability compared to Cronbach's alpha as it has more realistic underlying assumptions.^23^ Individual items were removed and the analysis re‐run if the internal consistency could be improved, although retaining as many items as possible. We judged an omega of 0.7 or higher to represent acceptable agreement. Following demonstrating adequate internal consistency, a summative score of these items were calculated (“Don't know” responses were classified as missing; cases with a missing item were excluded). To support interpretation, we also dichotomized the data to identify those who held the most negative (Strongly disagree or disagree responses across all items) and positive beliefs (Strongly agree or agree responses across all items).

A series of regression models between demographic factors and dementia attitude outcome. A multiple regression model was subsequently run with all independent variables in the model entered simultaneously.

The data were analyzed using IBM SPSS Statistics (version 27.0).^24^

## 3 RESULTS

A total of 4,413 individuals were included in the analysis. Participants were on average 58.0 years old (range 17–95 years old) and predominantly female (70.7%). There was roughly an equal split between people from Jakarta and North Sumatra. Approximately, two‐thirds of participants had completed at least a primary school level education.

Participants who were aware of the term's dementia and/or Alzheimer's tended to be younger, female, recruited from Jakarta, and to have completed primary education. See Table 1.

**TABLE 1:** Descriptive statistics of participants (total *n* = 4413)

		Missing (*n*)	Total		Not heard of dementia or Alzheimer's		Heard of dementia and/or Alzheimer's			
Characteristic			*M* (SD)	*N* (valid %)	*M* (SD)	*N* (valid %)	*M* (SD)	*N* (valid %)	Co‐eff	p
Age		9	57.99 (16.56)		59.32 (16.12)		49.72 (16.87)		*T* = 13.59	<0.001
Sex		9							*χ* ^2^ = 9.45	0.002
	Female			3118 (70.7%)		2656 (69.8%)		466 (75.9%)		
	Male			1295 (29.3%)		1147 (30.2%)		148 (24.1%)		
Site		10							*χ* ^2^ = 62.35	<0.001
	Jakarta			2218 (50.3%)		1818 (47.9%)		400 (65.0%)		
	North Sumatra			2194 (49.5%)		1979 (52.1%)		215 (35.0%)		
Schooling		48							*χ* ^2^ = 155.16	<0.001
	Less than primary			1029 (23.5%)		1108 (26.7%)		22 (3.6%)		
	Completed primary (or higher)			3345 (75.5%)		2765 (73.3%)		589 (96.4%)		

### 3.1 Terminology used by participants to describe dementia

A total of 3,803 people (86.3%) had not heard of the terms dementia and/or Alzheimer's disease before and were provided with a description of dementia. Overall, participants most commonly used the term *pikun* (*n* = 2873, 75.6%). Following content analysis, 3513 people (92.4%) provided responses that reflect cognitive impairment, and included terms related to forgetfulness, memory loss, or amnesia (e.g., *pikun, lupa, lupa ingatan, pelupa,amnesia, lali)*. *Pikun* also fits within this category. A total of 168 responses (4.4%) used terms associated with being dazed, confused, or absent minded (e.g., *linglung)*. Twenty‐one responses indicated uncertainty in what term they would use (0.6%). The remaining responses were composed of smaller numbers of terms (< 1%) related to, but not limited to, being crazy (e.g., *gila, kurang waras*), stupid (e.g., *Bodoh*), old age (e.g., *lansia*), stress (e.g., *stres*), and other health conditions (e.g., stroke, depression). Terms used tended to be in Bahasa Indonesian, although there were some that reflected slang and Indigenous languages (i.e., Batak, Javanese, Makassar, Sundanese, and Malay).

### 3.2 Dementia awareness and etiology beliefs

After adjusting for sociodemographic factors, participants that were aware of the dementia terms were more likely to believe that dementia was caused by brain disease, lack of family support, lifestyle, and genetics (*p* values < 0.05). People who had heard of the dementia terms were less likely to believe that dementia was caused by God's will, bad luck, and normal aging (*p* < 0.05). People who had not heard of the dementia terms were also more likely to respond that they did not know the cause. See Table 2.

**TABLE 2:** Odd ratios (and 95% CIs) of the association between dementia awareness in terminology and etiology beliefs (*n* = 4359)

	Never heard	Heard	Unadjusted		Adjusted^a^	
	Checked	Checked	OR	95% CIs	OR	95% CIs
Bad person	137 (3.7%)	22 (3.6%)	0.99	0.63–1.57	0.75	0.46–1.21
A brain disease	809 (21.6%)	239 (39.4%)	2.36	1.97–2.83	1.59	1.31–1.94
The way the person was raised	147 (3.9%)	40 (6.6%)	1.73	1.21–2.45	1.40	0.96–2.06
Genetics	499 (13.3%)	130 (21.5%)	1.78	1.43–2.20	1.29	1.02–1.63
Gods will	929 (24.8%)	156 (25.7%)	1.05	0.86–1.28	0.75	0.61–0.93
Bad luck	338 (9.0%)	35 (5.8%)	0.62	0.43–0.88	0.56	0.39–0.81
Normal aging	3030 (81.0%)	455 (75.1%)	0.71	0.58–0.87	0.62	0.50–0.77
Lifestyle	535 (14.3%)	185 (30.5%)	2.64	2.17–3.20	2.03	1.64–2.50
Witchcraft	193 (5.2%)	39 (6.4%)	1.27	0.89–1.81	0.83	0.57–1.21
Vaccinations	41 (1.1%)	5 (0.8%)	0.75	0.30–1.91	0.74	0.28–1.96
Stress	1643 (43.9%)	322 (53.1%)	1.45	1.22–1.72	1.09	0.90–1.31
Lack of family support	595 (15.9%)	151 (24.9%)	1.76	1.43–2.15	1.35	1.09–1.68
Other	224 (6.0%)	58 (9.6%)	1.66	1.23–2.25	1.64	1.19–2.25
Don't know	180 (4.8%)	11 (1.8%)	0.37	0.20–0.68	0.46	0.24–0.86

^a^ Adjusted for age, sex, education, and site.

### 3.3 Dementia attitude properties

Two‐items (*I would hide a diagnosis* and *I do not think people with dementia can contribute to society)* influenced the internal consistency of the attitudes scale sufficiently to warrant removal. The resulting seven‐item measure demonstrated adequate internal consistency (*ω* = 0.71, 95% CIs 0.69 to 0.73). On the summative scale, participants scored an average of 16.99 (standard deviation [SD] = 3.81; Min = 7, Max = 34; *n* = 3919). The skewness of the scale was 0.27, indicating the distribution approximately symmetric. When dichotomizing the items, only 18 participants (0.5%) disagreed (or strongly disagreed) with all statements, whereas 846 participants (21.6%) agreed (or strongly agreed) with all statements. Individual responses are reported in Table S1.

### 3.4 Dementia attitudes

People who had heard of dementia terms had fewer negative beliefs compared to those who had not heard of dementia. All participants who held universally negative beliefs about dementia (*n* = 18) had not previously heard of dementia terms before. Those who held universally positive beliefs, were more likely to have heard of dementia terms before. See Table 3.

**TABLE 3:** Description of dementia attitudes, split by those who have heard of the terms of dementia and/or Alzheimer's disease and those who have never heard of the terms

	Heard of dementia and/or Alzheimer disease (*n* = 563)		Never heard of terms (*n* = 3356)		Between group difference	
Parameter	*N* (valid%)	Mean ± SD	*N* (valid%)	Mean ± SD	Co‐efficient	95% CIs
Attitudes and beliefs (↑ more negative beliefs)		15.60 ± 3.68		17.22 ± 3.78	MD = 1.61	1.27–1.95
Universal responses						
Negative beliefs (disagree or strongly disagree to all items)	0 (0.0%)		18 (0.5%)		OR = 1.00	0.99–1.00
Positive beliefs (Agree or strongly agree to all items)	63 (29.0%)		683 (20.4%)		OR = 1.59	1.31–1.95

Adjusted analyses suggest that older age, having less than a primary school education and being recruited from North Sumatra were associated with more negative attitudes. Participants who believed dementia was a brain disease, had heard of the term Alzheimer's disease and/or dementia were less likely to hold negative attitudes. Being male was not associated with dementia attitudes. See Table 4.

**TABLE 4:** Multiple regression model of dementia attitudes

Parameter	*β*	*B*	95% CI	
Age	0.14	0.03***	0.02	0.04
Sex: Male	0.02	0.20	−0.06	0.45
Schooling: Primary and above	−0.04	−0.41**	−0.72	−0.11
Site: North Sumatra	0.11	0.85***	0.61	1.10
Dementia is a brain disease: Yes	−0.05	−0.41**	−0.69	−0.12
Heard of dementia and/or Alzheimer's disease	−0.09	−0.96***	−1.30	−0.61
Constant		15.19***	14.59	15.79

*Notes*: Higher scores represent more negative beliefs), with all variables entered simultaneously.

Associations statistically significant:*p < 0.05, **p < 0.01, ***p < 0.001.

## 4 DISCUSSION

This is the first study to explore public stigma toward dementia in Indonesia in a large purposive sample of adults. Our findings highlight that awareness and knowledge of dementia were limited, although attitudes were generally quite positive among the general public. Those with better knowledge and awareness tended to have better attitudes about dementia, but other sociodemographic factors also were associated with less stigmatizing attitudes.

Nearly two‐thirds of participants had not heard of the term dementia and/or Alzheimer's. In many cases, after providing a description of dementia, participants would use a term that reflected some form of cognitive impairment. Notably, *pikun* was by far the most used term. Colloquially, the term is used interchangeably with dementia (*demensia*).^21^ Official definitions for both terms extend past forgetfulness, although *demensia* explicitly refers to an underlying etiology (brain damage or disease), whereas *pikun* explicitly refers to old age.^25^ While there is no clear evidence that one term is more stigmatizing than the other, recognizing that dementia is a neurological disease might help shift away from the view that it is a normal part of aging. Labeling is a key feature of stigma, ^26^ and the choice of terminology can elicit implicit and explicit biases.^27^ Dementia and Alzheimer's disease are still labels, although have a pragmatic function of describing the condition or disease. Our finding that participants tended to adopt terms related to forgetfulness and memory loss could be appropriate, albeit reductionist, choice of terminology that is already in the general public's lexicon. Other countries have attempted to change stigmatizing terms of dementia.^28^ The extent to which these terms have been integrated into the countries lexicon and subsequently influenced stigma is unclear. It does appear that the present Japanese term “Ninchi‐sho” (neurocognitive disorder) does at least make family members of people with dementia feel less discomfort than that the previous term “Chiho” (silly or idiot).^29^ Certainly, the small minority of our sample who adopted terms related to being crazy or stupid is concerning.

The lack of adoption of clinically appropriate terminology among the general public could be inferred that there is a lack of awareness and knowledge about the condition. We demonstrate that people who had heard of these terms were more likely to have an accurate biomedical understanding of dementia etiology (e.g., it is a brain disease), while those unaware of the terms tended to hold misconceptions (e.g., it is normal aging). Believing dementia was a normal part of aging was the most commonly selected etiology, thus reflecting how common the belief is internationally.^4^ The large numbers of people who were unaware of the terms and were unable to identify appropriate etiology of the condition, does suggest that any potential public awareness campaigns have been ineffective at least in increasing knowledge. While we did not explicitly ask about previous access of information, and a broader set of knowledge based questions, our findings appear not to align with evidence that the majority of Indonesian adults (70%) have received information about dementia in the past.^30^ However, the findings from Susilowati and colleagues should be interpreted with caution due to their being derived from an opportunistic sample of middle‐aged adults from social media. The exact reason why certain groups (e.g., North Sumatran, males) are less likely to be aware of these terms are unclear; however, it does highlight potential targets for awareness and education campaigns.

Our findings demonstrate that for the most part, people held positive or mixed attitudes toward dementia, and only a very small minority held universally negative attitudes. Exploration of individual items does indicate that a sizeable minority held negative attitudes on specific issues, with a quarter of participants not seeing a value in a formal diagnosis (*n* = 1118). As attitudes help inform behaviors, this has potential implications about how behaviors might manifest in Indonesia. For example, beliefs that dementia does not have a medical etiology, may lead to attitudes that reflect nihilism to a diagnosis and treatment, which in turn drives people not to a seek a diagnosis. Within our own research, very few older adults had reported to have received a formal diagnosis (0.2%).^3^ While this could be due to inadequate services, it may also represent a reluctance or lack of knowledge to seek a diagnosis.

In addition to having poorer awareness of the condition, older adults and participants with little to no formal education were more likely to hold negative attitudes. Such demographics have been previously found to be associated with attitudes or reactions toward dementia in the broader literature in other settings.^31^, ^32^, ^33^ Unlike some other literature,^34^, ^35^ males did not have poorer attitudes. While it is unclear why attitude differences exist between North Sumatra and Jakarta, our findings further highlight that we should not assume dementia stigma looks the same everywhere.

Underlying awareness and knowledge of the condition was associated with better attitudes and beliefs. These findings are perhaps unsurprising as education and awareness are one of the key strategies to tackle stigma.^18^, ^36^ Having heard of either dementia and/or Alzheimer's disease was also found to be associated with better attitudes. The association remained even after accounting for accurate etiology beliefs in the model (i.e., it is a brain disease), indicating that the association is not wholly due to understanding the biomedical model of dementia.

There are several limitations to consider. First, we should be vigilant that we did not confirm that people who had previously heard of dementia and/or Alzheimer's disease did in fact correctly understand what they meant. Second, we are only able to provide limited psychometric properties about our measures of attitudes and beliefs. Future research should use validated questionnaires. Third, the inclusion of those who had only just been introduced the terms dementia and Alzheimer's disease, despite commonly adopting colloquial terms in its place, may introduce bias to our analysis. If this was the case, we might expect a propensity for participants to select more neutral responses (e.g., “Don't know” and “neither agree nor disagree”), but that does not appear to be the case. Fourth, our randomized sampling strategy is likely to have facilitated a more representative cohort of older adults; however, as the informants were sampled by convenience (in that they knew the older adult well) we are unable to make the same assumptions. Fifth, the outcome measure incorporated items related to attitudes toward diagnosis and treatment, as well as items related to living well with dementia. We are unable to confirm whether the associations reported here are universal across all dementia attitudes, or maybe driven by a subset of items. Finally, the outcomes only incorporate certain elements of public stigma (e.g., knowledge, beliefs) and do not contain others (e.g., discrimination).^6^ Discrimination is particularly important because it represents the behavioral manifestation of stigma, thus, can have profound impact on the lives of people living with dementia.

The majority of Indonesian adults held mixed or positive attitudes toward dementia, despite widespread lack of awareness and misconceptions. Raising awareness about dementia could be a key strategy to improve these attitudes. Through recognizing that negative attitudes are more prevalent in certain subgroups, we can perhaps better optimize the delivery of these strategies.

## CONFLICT OF INTEREST STATEMENT

None to declare. Author disclosures are available in the supporting information.

## CONSENT STATEMENT

Informed consent was obtained (written or oral) from the older adult and an identified informant.

## Supporting information

Supporting Information

ICMJE Disclosure Form

## Data Availability

Data will become available on UK Data Services in 2024 and is available from the corresponding author (N.F) upon reasonable request.

## Footnotes

a We recognize that there can be great variation in the symptoms experienced by someone with dementia, and that dementia can occur in younger adults.
