# Supplementary material for: Awareness, attitudes, and beliefs of dementia in Indonesia
Source: Alzheimers Dement (Amst). 2024 Apr 12;16(2):e12570. doi: 10.1002/dad2.12570 (PMC11010264; doi:10.1002/dad2.12570)
Supplement: Supplementary file 1 — Supporting Information [file DAD2-16-e12570-s002.docx]

**Supplementary Table 1: Individual responses to attitude items**

|  |  |  |  |  |
| --- | --- | --- | --- | --- |
|  |  |  |  |  |
| Item | Response |  | N | % |
| A person with dementia’s memory will improve with treatment. | Strongly Agree |  | 209 | 4.7 |
|  | Agree |  | 1619 | 36.5 |
|  | Neither agree nor disagree |  | 390 | 8.8 |
|  | Disagree |  | 1697 | 38.3 |
|  | Strongly Disagree |  | 253 | 5.7 |
|  | Don’t know |  | 226 | 5.1 |
|  | Missing |  | 38 | 0.9 |
|  |  |  |  |  |
|  |  |  |  |  |
| There is value in a person with dementia being given a formal diagnosis from a doctor. | Strongly Agree |  | 255 | 5.8 |
|  | Agree |  | 2365 | 53.4 |
|  | Neither agree nor disagree |  | 466 | 10.5 |
|  | Disagree |  | 992 | 22.4 |
|  | Strongly Disagree |  | 126 | 2.8 |
|  | Don’t know |  | 188 | 4.2 |
|  | Missing |  | 40 | 0.9 |
|  |  |  |  |  |
|  |  |  |  |  |
| We can do a lot now to improve the lives of people with dementia. | Strongly Agree |  | 363 | 8.2 |
|  | Agree |  | 2746 | 62.0 |
|  | Neither agree nor disagree |  | 395 | 8.9 |
|  | Disagree |  | 682 | 15.4 |
|  | Strongly Disagree |  | 63 | 1.4 |
|  | Don’t know |  | 141 | 3.2 |
|  | Missing |  | 42 | 0.9 |
|  |  |  |  |  |
| People with dementia can enjoy life. | Strongly Agree |  | 310 | 7.0 |
|  | Agree |  | 2102 | 47.4 |
|  | Neither agree nor disagree |  | 463 | 10.4 |
|  | Disagree |  | 1223 | 27.6 |
|  | Strongly Disagree |  | 129 | 2.9 |
|  | Don’t know |  | 162 | 3.7 |
|  | Missing |  | 43 | 1.0 |
|  |  |  |  |  |
|  |  |  |  |  |
| We need to be more tolerant toward people with dementia in our society. | Strongly Agree |  | 592 | 13.4 |
|  | Agree |  | 3364 | 75.9 |
|  | Neither agree nor disagree |  | 191 | 4.3 |
|  | Disagree |  | 192 | 4.3 |
|  | Strongly Disagree |  | 11 | 0.2 |
|  | Don’t know |  | 43 | 1.0 |
|  | Missing |  | 39 | 0.9 |
|  |  |  |  |  |
|  |  |  |  |  |
|  |  |  |  |  |
| I do not think that people with dementia can make a positive contribution to society. | Strongly Agree |  | 183 | 4.1 |
|  | Agree |  | 2041 | 46.1 |
|  | Neither agree nor disagree |  | 361 | 8.1 |
|  | Disagree |  | 1540 | 34.7 |
|  | Strongly Disagree |  | 152 | 3.4 |
|  | Don’t know |  | 110 | 2.5 |
|  | Missing |  | 45 | 1.0 |
|  |  |  |  |  |
|  |  |  |  |  |
| If I had dementia, I would hide the diagnosis from others. | Strongly Agree |  | 129 | 2.9 |
|  | Agree |  | 998 | 22.5 |
|  | Neither agree nor disagree |  | 192 | 4.3 |
|  | Disagree |  | 2498 | 56.4 |
|  | Strongly Disagree |  | 515 | 11.6 |
|  | Don’t know |  | 57 | 1.3 |
|  | Missing |  | 43 | 1.0 |
|  |  |  |  |  |
|  |  |  |  |  |
|  |  |  |  |  |
| If I saw someone with dementia struggling to do something, I would help them. | Strongly Agree |  | 645 | 14.6 |
|  | Agree |  | 3468 | 78.2 |
|  | Neither agree nor disagree |  | 142 | 3.2 |
|  | Disagree |  | 97 | 2.2 |
|  | Strongly Disagree |  | 5 | 0.1 |
|  | Don’t know |  | 34 | 0.8 |
|  | Missing |  | 41 | 0.9 |
|  |  |  |  |  |
| If I suspected that I had dementia, I would go to a health professional for help. | Strongly Agree |  | 384 | 8.7 |
|  | Agree |  | 2842 | 64.1 |
|  | Neither agree nor disagree |  | 214 | 4.8 |
|  | Disagree |  | 819 | 18.5 |
|  | Strongly Disagree |  | 69 | 1.6 |
|  | Don’t know |  | 55 | 1.2 |
|  | Missing |  | 49 | 1.1 |
|  |  |  |  |  |
